# Targeting Acid Ceramidase Inhibits Glioblastoma Cell Migration through Decreased AKT Signaling

**DOI:** 10.3390/cells11121873

**Published:** 2022-06-09

**Authors:** Cyntanna C. Hawkins, Amber B. Jones, Emily R. Gordon, Sarah E. Williford, Yuvika Harsh, Julia K. Ziebro, Catherine J. Landis, Sajina Gc, David K. Crossman, Sara J. Cooper, Sasanka Ramanadham, Ninh Doan, Anita B. Hjelmeland

**Affiliations:** 1 Department of Cell, Developmental and Integrative Biology, University of Alabama at Birmingham, Birmingham, AL 35233, USA; cyntanna@uab.edu (C.C.H.); amberj96@uab.edu (A.B.J.); sarah24@uab.edu (S.E.W.); yharsh14@uab.edu (Y.H.); cat.landis14@gmail.com (C.J.L.); sajinagc@uab.edu (S.G.); sramvem@uab.edu (S.R.); 2 HudsonAlpha Institute for Biotechnology, Huntsville, AL 35806, USA; egordon@hudsonalpha.org (E.R.G.); sjcooper@hudsonalpha.org (S.J.C.); 3 Graduate Biomedical Sciences, Division of Neuropathology, Department of Pathology, O’Neal Comprehensive Cancer Center, University of Alabama School of Medicine, Birmingham, AL 35233, USA; ziebroj1@uab.edu; 4 Department of Genetics, University of Alabama at Birmingham, Birmingham, AL 35294, USA; dkcrossm@uab.edu; 5 Comprehensive Diabetes Center, University of Alabama at Birmingham, Birmingham, AL 35294, USA; 6 Baptist South Medical Center, Montgomery, AL 36116, USA; ninhdoan79@gmail.com

**Keywords:** glioblastoma, acid ceramidase, migration, ceramides, AKT

## Abstract

Glioblastoma (GBM) remains one of the most aggressive cancers, partially due to its ability to migrate into the surrounding brain. The sphingolipid balance, or the balance between ceramides and sphingosine-1-phosphate, contributes to the ability of GBM cells to migrate or invade. Of the ceramidases which hydrolyze ceramides, acid ceramidase (ASAH1) is highly expressed in GBM samples compared to non-tumor brain. ASAH1 expression also correlates with genes associated with migration and focal adhesion. To understand the role of ASAH1 in GBM migration, we utilized shRNA knockdown and observed decreased migration that did not depend upon changes in growth. Next, we inhibited ASAH1 using carmofur, a clinically utilized small molecule inhibitor. Inhibition of ASAH1 by carmofur blocks in vitro migration of U251 (GBM cell line) and GBM cells derived from patient-derived xenografts (PDXs). RNA-sequencing suggested roles for carmofur in MAPK and AKT signaling. We found that carmofur treatment decreases phosphorylation of AKT, but not of MAPK. The decrease in AKT phosphorylation was confirmed by shRNA knockdown of ASAH1. Our findings substantiate ASAH1 inhibition using carmofur as a potential clinically relevant treatment to advance GBM therapeutics, particularly due to its impact on migration.

## 1. Introduction

Dysregulated sphingolipid metabolism is a common feature of glioblastoma (GBM), the most common primary malignant brain tumor with a median survival of only 14–16 months [1,2]. Despite maximal safe surgical resection, radiotherapy, or chemotherapy with temozolomide (TMZ), GBM almost always recurs, contributing to the dismal prognosis [3]. Recent work demonstrated that after radiotherapy, GBM patients have elevated levels of acid ceramidase (ASAH1) protein. The ASAH1 belongs to a family of ceramidases that are involved in sphingolipid metabolism [4]. Specifically, they hydrolyze pro-apoptotic ceramides to sphingosine, which can be phosphorylated by sphingosine kinases to produce sphingosine-1-phosphate (S1P). S1P plays a critical role in cell evasion of apoptosis [5]. Contributing to the highly invasive characteristics of GBM, S1P has been shown to increase migration of GBM cell lines when added to migration assays in culture [6]. Importantly, TCGA heatmap analysis suggests that *ASAH1* is the only ceramidase that is expressed highly in the GBM tumor, as compared to non-tumor brain [7].

Inhibitors of acid ceramidase have shown promise in vitro by inducing apoptosis and increasing ceramides [8,9]. Some ASAH1 inhibitors, including ARN14988, N-oleoylethanolamine, and carmofur, have been shown to decrease the growth of GBM cell lines and cells isolated from GBM patient-derived xenografts (PDX) [8]. These studies emphasize the role of ASAH1 on GBM cell survival, yet, none of the *ASAH1* inhibitors have reached clinical trials for GBM. Since the blood-brain barrier would likely limit success of many of the currently established inhibitors of ASAH1, we chose to focus on carmofur as it is known to cross the blood–brain barrier and has been used clinically in Japan to treat colorectal cancer since the 1980s [9,10,11,12]. Carmofur, a derivative of 5-fluorouracil (5-FU), covalently binds to the active site of ASAH1 to inhibit its function [13]. In contrast to 5-FU, which did not alter ASAH1 activity or levels of ceramides, in vitro studies in adenocarcinoma cell lines showed that carmofur increased ceramides, specifically C14:0, C16:0, and C18:0 [9]. The C18:0 ceramide, produced by ceramide synthase 1, is the most abundant ceramide in the brain and is decreased in GBM patient samples compared to non-tumor brain [1,14]. C18:0 leads to the dephosphorylation of many oncogenic proteins such as AKT and ERK by activating protein phosphatase-1 (PP1A) and protein phosphatase-2A (PP2A) [15]. In GBM, ASAH1 expression is associated with CD133^+^ GBM cells, a population of stem-like GBM cells which are highly invasive [8,16] and particularly sensitive to AKT inhibition leading to decreased growth and migration [17].

Shifting the balance from S1P to ceramides via ASAH1 inhibition could serve as a mechanism to overcome GBM cell migration. Though an ability of ASAH1 inhibition in blocking invasion of melanoma cells has been demonstrated [18], the effects on GBM cell migration of genetic targeting or pharmacologic inhibition of ASAH1 with carmofur have not been determined. Here, we established a novel role for carmofur in the inhibition of GBM cell migration and identified loss of AKT phosphorylation as a mechanism for this phenotype.

## 2. Materials and Methods

### 2.1. Cell Lines

The patient-derived xenografts (PDXs) D456 and JX22 were gifts from Dr. Darrel Bigner (Duke University) and Dr. Jann Sarkaria (Mayo Clinic; https://www.mayo.edu/research/labs/translational-neuro-oncology/mayo-clinic-brain-tumor-patient-derived-xenograft-national-resource/pdx-characteristics/pdx-phenotype) (accessed on 25 February 2021), respectively. GBM PDX were propagated as subcutaneous xenografts in Balbc nu/nu mice initiated from minced PDX tissue or cells, in accordance with the guidelines established by UAB Institutional Animal Care and Use Committee. Once xenografts were approximately 10 mm in diameter, xenografts were dissociated as we and others have previously described using a Papain Dissociation System (Worthington Biochemical Corporation, Lakewood, NJ, USA) [16,17,19]. Isolated cells were maintained in culture for a maximum of 10 passages to preserve their molecular features [20,21]. The U251 cell line was obtained from Dr. Corinne Griguer (University of Iowa, previously at UAB).

### 2.2. Culture of Cell Lines and Patient-Derived Xenografts

All cells isolated from GBM PDX and cell lines were cultured in the absence of serum, under conditions which more faithfully recapitulate the molecular and cellular heterogeneity of GBM patients [22,23]. Media consist of DMEM/F12 (Life Technologies, Carlsbad, CA, USA, cat# 21041-025) supplemented with Gem21 Neuroplex without vitamin A (Gemini Bioproducts, West Sacramento, CA, USA, cat# 400-161), 100 U/mL penicillin and 100 µg/mL streptomycin (Gibco, Waltham, MA, USA, cat# 15-140-122), 1% sodium pyruvate (Gibco, Waltham, MA, USA, cat# 11360070), and 10 ng/mL of epidermal growth factor and fibroblast growth factor (Gemini Bioproducts, West Sacramento, CA, USA, cat# 300-110P and 300-112P). Where indicated for short-term experiments requiring monolayer of cells or in which phosphorylation was stimulated, FBS media consisted of DMEM/F12 with 10% FBS (Peak Serum, Wellington, CO, USA, cat# PS-FB2), 100 U/mL penicillin and 100 µg/mL, and 1% sodium pyruvate. Propagation of CSC293T cells for generation of lentivirus was conducted as we described [24].

### 2.3. Accession and Analysis of Publicly Available Datasets

The Cancer Genome Atlas (TCGA) and Gravandeel datasets were accessed using the GlioVis portal (http://gliovis.bioinfo.cnio.es) (accessed on 25 February 2021) [8]. Histology graphs were compared using either independent t-test or one-way ANOVA followed by Dunnett’s multiple comparisons test, where applicable. Survival was analyzed using Kaplan–Meier survival curves followed by log-rank test. Optimal cutoff was used in GlioVis to determine high and low expression of the relevant genes unless otherwise stated. Gene correlations >0.3 and <−0.3 to *ASAH1* were also downloaded from GlioVis and uploaded to WebGestalt (http://www.webgestalt.org) (accessed on 12 August 2021) [25] for gene set enrichment analysis (GSEA) compared to WikiPathway Cancer with a minimum of 10 genes per pathway and weighted set cover to reduce redundancy of pathways.

### 2.4. Generation of Knockdown Cell Lines

Knockdown cells were generated by lentiviral infection using a non-targeting control for the PLKO.1 vector (Millipore Sigma, St. Louis, MO, USA, cat# SHC001) or Precision LentiORF positive control (Horizon Discovery, Cambridge, UK, cat# OHS5832) as a fluorescent positive control for infection and two different ASAH1-directed shRNA constructs (Millipore Sigma, St. Louis, MO, USA, cat# TRCN0000235585 and TRCN0000219048). Constructs were transfected into CSC293T cells using Fugene HD (Promega, Madison, WI, USA, cat# PRE2312). After 48, 72, and 96 h, virus was collected for infection. Viral RNA was isolated using NucleoSpin RNA (Takara Bio, San Jose, CA, USA, cat# 740956.50) and titered using the Lenti-X™ qRT-PCR Titration Kit (Takara Bio, San Jose, CA, USA, cat# OHS6085). To infect GBM cells, a multiplicity of infection of 10 was used. For migration assays, infected GBM cells were growth factor-deprived 24 h after infection. GBM cells were collected and plated for experiments 48 h after infection. For each infection, RNA was collected at the time of plating for the experiment to confirm knockdown.

### 2.5. RNA Isolation and Quantitative Real-Time PCR

RNA was isolated using the Qiagen RNA isolation kit (Germantown, MD, USA, cat# 74106) according to the manufacturer’s protocol. cDNA was generated from RNA using the iScript cDNA synthesis reaction (BioRad, Hercules, CA, USA, cat# 170-8891). SsoAdvanced Universal SYBR Green Supermix (BioRad, Hercules, CA, USA, cat# 172-5274) was used for RT-qPCR. Additional controls included “no template” and “no reverse transcriptase” groups for each primer tested. Delta CT values were determined with normalization to β-actin values. Human *ASAH1* primer (qHsaCID0014767) was purchased from BioRad (Hercules, CA, USA) and is known to be intron-spanning based on their validation. Primers for human *β-ACTIN* were bought from IDT (Coralville, IA, USA) with sequences as follows: *β-ACTIN* FWD: AGA AAA TCT GGC ACC ACA CC; *β-ACTIN* REV: AGA GGC GTA CAG GGA TAG CA.

### 2.6. Cell Growth Assays

Cells were plated at a density of 1000–5000 cells per well of a 96-well plate and allowed to recover overnight. ASAH1 inhibitors, carmofur (Selleckchem, Houston, TX, USA, cat# S1289), B13 (Cayman Chemical, Ann Arbor, MI, USA, cat# 10006305), or N-oleoylethanolamide (Cayman Chemical, Ann Arbor, MI, USA, cat# 90265) were added at concentrations between 5 µM and 100 µM as indicated in figure legends. After the indicated times, Cell Titer Glo Assay was completed as directed by the manufacturer (Promega, Madison, WI, USA).

### 2.7. Scratch Assays

Cells were plated at a density of 100,000 cells per well of a 24-well plate in FBS media to allow cells to adhere. After cells proliferated to form a confluent monolayer, they were serum-starved overnight in the presence of vehicle (DMSO), carmofur, or B13 at the indicated concentrations. The following day, a scratch was made in the shape of an ‘X’ using a P1000 pipet tip with fresh FBS media and vehicle, B13, or carmofur. The scratch was imaged in the same location at the indicated times. At the conclusion of the scratch assay, cells were fixed in 10% formalin and stained with 0.5% crystal violet. Image centers of 24 h 20×images were quantified using particle analysis in ImageJ and normalized to 0 h images before normalizing to vehicle for quantification.

### 2.8. Boyden Chamber Assays

Cells were growth factor-deprived overnight in the presence of carmofur, as indicated in figure legends. Cells were then counted and seeded at a density of 50,000 cells per 250 µL in the upper chamber of the Boyden chamber (Corning, Corning, NY, USA cat# 353097) with 8 µm pores. At time of plating, fresh carmofur was added with the cells in the upper chamber. The lower chamber contained the media with chemoattractant: 10% FBS media or DMEM/F12 supplemented with Gem21 Neuroplex without vitamin A, 1% penicillin/streptomycin, 1% sodium pyruvate, 20 ng/mL of epidermal growth factor and fibroblast growth factor (2× EFG/FGF), and 2% FBS. After 4–8 h, cells that migrated through to the underside of the membrane were fixed in 10% formalin, stained with 0.5% crystal violet, and quantified using the cell count function of the Pico Imagepress (Molecular Devices, San Jose, CA, USA) or Particle analysis in ImageJ.

### 2.9. RNA-Sequencing Analysis

RNA from cell pellets was extracted using Norgen Total RNA following the manufacturer’s protocol (Thorold, ON, USA, cat# 37500, 25710). RNA quality was verified with the Agilent BioAnalyzer RNA Nano 600 kit (Santa Clara, CA, USA, cat# 5067-1512) with the RIN range between 9–10. RNA-sequencing libraries were made using Lexogen QuantSeq 3’ mRNA-Seq Library Prep Kit FWD for Illumina kit (Vienna, Austria, cat# 015.24) with 250 ng of RNA input. They were pooled and sequenced on an Illumina NextSeq 500 instrument with 75 bp single end reads. Read counts averaged 11.2 million reads and average Q30% was 94.28. Lexogen’s BlueBee integrated QuantSeq data analyses pipeline was used for trimming, mapping, and alignment, and DESeq2 implemented in R was used for determination of differential expression [26]. All differentially expressed genes with a base mean > 5, log_2_ fold change of < −0.5 or > 0.5, and *p* value < 0.2 (unadjusted) were analyzed using WebGestalt (http://www.webgestalt.org) (accessed on 4 December 2021) [25] for gene set enrichment analysis and Qiagen Ingenuity Pathway Analysis (https://digitalinsights.qiagen.com/IPA) (accessed on 18 February 2022) [27]. Data for RNA-sequencing analysis can be found on the Gene Expression Omnibus with the accession number GSE179087.

### 2.10. Lysate Preparation and Immunoblot Analysis

For phosphorylation analysis, cells were plated at a density of 1 × 10^6^ cells per 100 mm plate in FBS media. The following day, cells were serum-starved in the presence of 5 µM carmofur for 16 h. After serum starvation, cells were pre-treated with a final concentration of 10% FBS for 15 min followed by carmofur treatment for 45 min. For ASAH1 KD phosphorylation analysis, cells were transduced 48 h prior to being serum-starved followed by a 1 h stimulation with 10% FBS. Then, cells were washed with PBS (ThermoFisher Scientific, Waltham, MA, USA, cat# 10010049) and lysed directly with RIPA Lysis and Extraction Buffer (ThermoFisher, Waltham, MA, USA, cat# 89901) plus Halt protease and phosphatase inhibitors (Invitrogen, Waltham, MA, USA, cat# 78440). Lysate was passaged through a 28G needle and spun down at 15,000 rpm for 15 min before quantification using the BCA protein assay (Fisher Scientific, Waltham, MA, USA, cat# 23227). Samples were prepared in Pierce Lane Marker Reducing 5× Sample Buffer (ThermoFisher, Waltham, MA, USA, cat# 39000) with 60 µg of protein. Gel electrophoresis used Novex Wedge Well 4–20% Tris-Glycine gels (Invitrogen, Waltham, MA, USA, cat# xp04200Box) followed by transfer to PVDF membranes (Fisher Scientific, Waltham, MA, USA, cat# SLHV033RS). Blocking was achieved using protein-free T20 (TBS) blocking buffer (Thermo Scientific, Waltham, MA, USA, cat#37571) for 1 h at room temperature. Primary antibodies were incubated overnight at 4 °C followed by incubation of secondary antibodies at room temperature for 1 h with IRDye 680CW goat anti-mouse IgG or IRDye 800CW goat anti-rabbit IgG (LI-COR Biosciences, Lincoln, NE, USA, cat# 926-68070 and 926-32211). The following primary antibodies were used for immunoblotting: Phospho-AKT (Ser473, Cell Signaling, Danvers, MA, USA, cat# 9271), total AKT (Cell Signaling, Danver, MA, USA, cat# 9272), phospho-ERK 1/2 (Thr202/Tyr204, Thr185/Tyr187, Millipore Sigma, St. Louis, MO, USA, cat # 05-797R), total ERK 1/2 (Cell Signaling, Danvers, MA, USA, cat# 9102), phospho-FAK (Tyr397, Cell Signaling, Danvers, MA, USA, cat #3283) ASAH1 (Origene, Rockville, MD, USA, cat# AM31723PU-N) and β-Actin loading control antibody (Invitrogen, Waltham, MA, USA, cat# MA5-15739). Blots were imaged using an Odyssey infrared imaging system (LI-COR Biosciences, Lincoln, NE, USA). Densitometry values for each protein were determined using Image Studio Lite v5.2 software (LI-COR Biosciences, Lincoln, NE, USA).

### 2.11. Statistical Analysis

All experiments were completed in biological triplicates with a minimum of three technical replicates per experiment. Analyses of data were completed using Prism v9 (Graphpad Software, San Diego, CA, USA) with relevant tests listed in the figure legends.

## 3. Results

### 3.1. ASAH1 Expression Correlated with Worse Survival in GBM Patients

**Figure 1:**
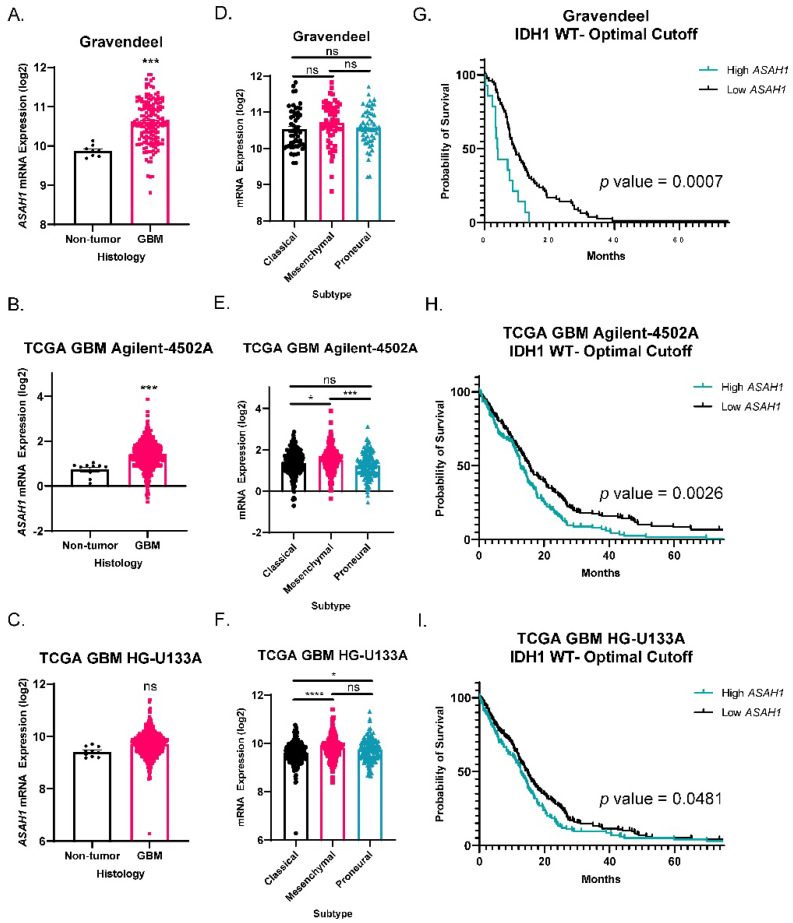
*ASAH1* correlated with worse survival in IDH1 wildtype GBM patients. Datasets were accessed using GlioVis (http://gliovis.bioinfo.cnio.es) (accessed on 25 February 2021). *ASAH1* mRNA expression in GBM compared to non-tumor samples in (**A**) Gravandeel (*n* = 8 for non-tumor, *n* = 159 for GBM), (**B**) TCGA GBM Agilent-4502A platform (*n* = 10 for non-tumor, *n* = 489 for GBM), and (**C**) TCGA GBM HG-U133A platform (*n* = 10 for non-tumor, *n* = 528 for GBM). Data were analyzed by independent *t*-test. *ASAH1* mRNA expression in the three subtypes of GBM (**D**) Gravandeel (*n* = 52 for classical, *n* = 54 for mesenchymal, *n* = 53 for proneural), (**E**) TCGA GBM Agilent-4502A platform (*n* = 182 for classical, *n* = 156 for mesenchymal, *n* = 121 for proneural), and (**F**) TCGA GBM HG-U133A platform (*n* = 199 for classical, *n* = 166 for mesenchymal, *n* = 163 for proneural). Data were analyzed by one-way ANOVA followed by Dunnett’s multiple comparisons test and displayed as means ± SEM. Kaplan–Meier survival curves for IDH1 wildtype GBM patients using optimal cutoff to determine high and low *ASAH1* expression. (**G**) Gravandeel (*n* = 93), (**H**) TCGA GBM Agilent-4502A platform (*n* = 339), and (**I**) TCGA GBM HG-U133A platform (*n* = 372). Groups were compared by log-rank test. Not significant (ns), * *p* < 0.05, *** *p* < 0.001, **** *p* < 0.0001.

To understand the expression pattern of *ASAH1* in GBM as compared to non-tumor brain, we evaluated *ASAH1* gene expression in three different publicaly available patient mRNA datasets. *ASAH1* levels were significantly higher in GBM tissue compared to non-tumor brain in Gravandeel and TCGA GBM Agilent-4502A (Figure 1A,B) and similarly trended higher in TCGA GBM HG-U133A (*p* = 0.0516) (Figure 1C). Next, we assessed *ASAH1* mRNA among three major GBM subtypes. Proneural, mesenchymal, and classical GBM subtypes are known to involve differences in PDGFRA amplification, NF1 loss, and EGFR amplification, respectively [28]. While the mesenchymal subtype, which is known for its highly invasive and migratory phenotype [29], had significantly higher *ASAH1* mRNA in the TCGA GBM Agilent-4502A dataset (Figure 1E), consistently signficant difference in *ASAH1* mRNA levels were not detected across subtypes among the datasets (Figure 1D,F). Lastly, we assessed survival of IDH1-wildtype GBM patients based on *ASAH1* expression. IDH1-wildtype status is required for GBM designation [30], and we selected these samples for the initial analysis to ensure any survival differences were not due to associations with glioma grade or IDH status. High *ASAH1* mRNA correlated with a shorter median survival in all three databases for IDH1-wildtype GBM patients (Figure 1G–I). Elevated *ASAH1* mRNA was also significantly associated with worse survival for all glioma patients (GBM and low-grade gliomas) (Supplementary Figure S1A). In contrast, there was no signficant correlation of elevated expression of other ceremidases (*ASAH2*, *ACER2*, *ACER3*) with poor glioma patient outcomes (Supplementary Figure S1B–D).

### 3.2. ASAH1 Expression Correlated with Genes in the Focal Adhesion-PI3K-AKT-mTOR Pathway

**Figure 2:**
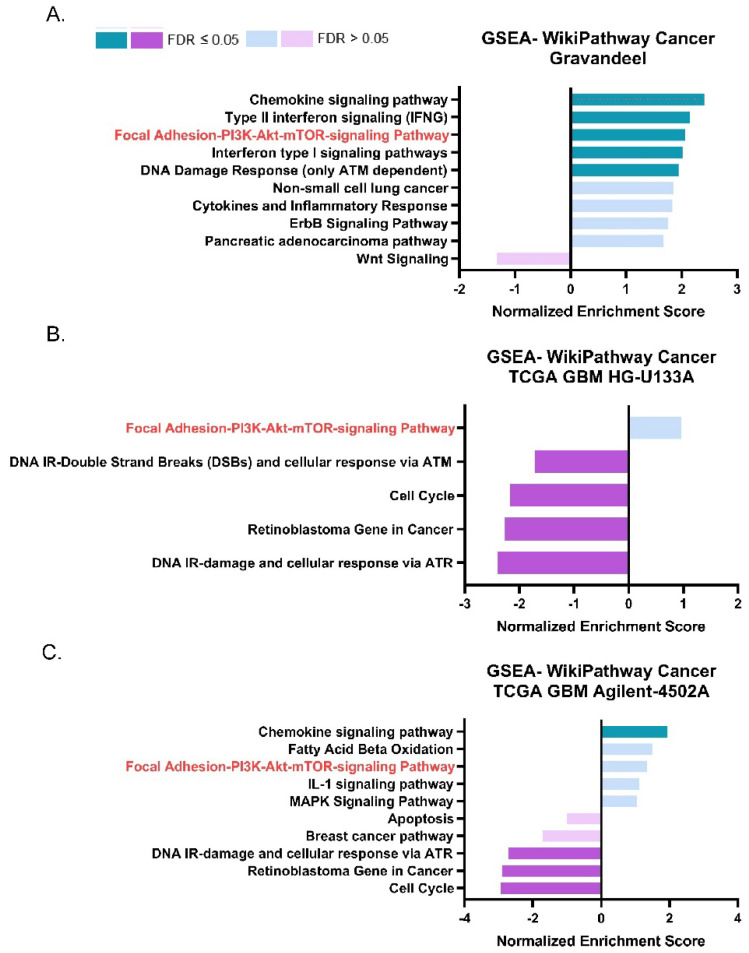
*ASAH1* expression positively correlated with genes in the Focal Adhesion-PI3K-AKT-mTOR pathway. Pathway analysis of positive and negative gene correlations to *ASAH1*. Datasets were accessed using GlioVis (http://gliovis.bioinfo.cnio.es) (accessed on 12 August 2021). GSEA of *r* values > 0.3 and <−0.3 for genes compared to *ASAH1* in (**A**) Gravandeel (**B**) TCGA GBM HG-U133A, and (**C**) TCGA GBM Agilent-4502A. Data were analyzed using WebGestalt (http://www.webgestalt.org) (accessed on 12 August 2021) and compared to Wikipathway cancer with a 10 gene minimum/pathway and weighted set cover.

We next sought to identify pathways and phenotypes associated with *ASAH1* expression in GBM patients. We performed gene set enrichment analysis (GSEA) using WebGestalt, a web-based gene set analysis toolkit [25], to identify pathways that have a high degree of positive (>0.3) or negative (<−0.3) correlation with *ASAH1*. This analysis was performed using the same publicly available datasets as in Figure 1. The focal adhesion-PI3K-Akt-mTOR-signaling pathway positively correlated with *ASAH1* in the Gravandeel dataset (Figure 2A) and had a positive normalized enrichment score in both TCGA GBM HG-U133A (Figure 2B) and TCGA GBM Agilent-4502A (Figure 2C). This signaling pathway was the only pathway which appeared in all three GSEA of the datasets, although we only observed a statistically significant enrichment with low false discovery rate in the Gravandeel dataset (Table 1). Furthermore, *ASAH1* expression positively correlated with an increase in chemokine signaling in two of the datasets which further emphasizes the utility of this analysis: S1P is a known chemokine for immune populations such as macrophages and T cells [31,32] (Supplementary Figure S2). In view of the association of *ASAH1* expression with the focal adhesion-PI3K-AKT-mTOR pathway and the known link between ceremide levels and AKT signaling [33,34], we focused on the potential biological role of *ASAH1* in GBM cell migration.

### 3.3. ASAH1 Knockdown Decreased Migration of GBM Cells Derived from PDXs

**Figure 3:**
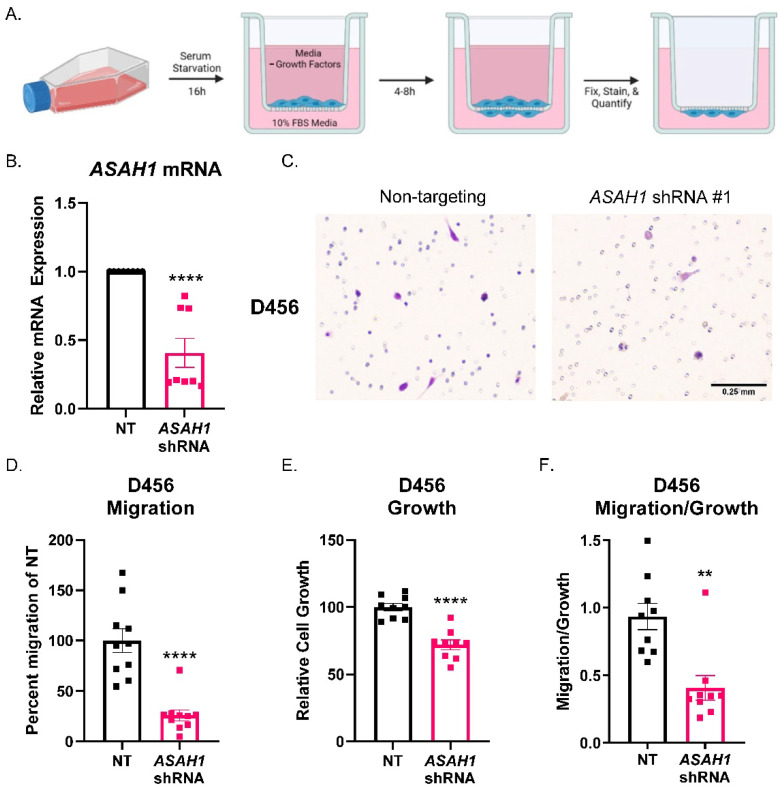
ASAH1 knockdown decreased migration of D456 cells. Cells were transduced with either non-targeting (NT) control or ASAH1 shRNA. After 48 h, the cells were plated at 50,000 cells/insert for the Boyden chamber migration assay with 10% FBS as a chemoattractant, 5000 cells/well for growth assay, and the remaining for RNA. (**A**) Schematic showing Boyden chamber assay steps. (**B**) mRNA expression of *ASAH1* at time of assay normalized to β-Actin using ∆∆CT. (**C**) Representative images of D456 migration inserts with crystal violet staining for non-targeting and ASAH1-directed shRNA constructs. (**D**) Migration inserts were imaged with 6 images at 10× magnification/insert and quantified using particle analysis in ImageJ and normalized to the NT control. (**E**) Matching growth was analyzed by Cell Titer Glo and normalized to NT control. (**F**) Percent migration was divided by the relative cell growth to determine the ratio of migration to growth. Data from three independent experiments were combined and analyzed using independent *t*-test (*n* = 3–4 per experiment). Data are displayed as means ± SEM. ** *p* < 0.01, **** *p* < 0.0001.

To determine the effect of ASAH1 on GBM cell migration, we utilized ASAH1-targeted shRNA and analyzed migration in Boyden chamber assays (Figure 3A) using D456 GBM cells isolated from the patient-derived xenograft (PDX). The D456 cells represent the proneural subtype of GBM, and we previously demonstrated their invasive properties [35]. *ASAH1* mRNA was significantly decreased when cells were transduced with lentivirus expressing ASAH1-directed shRNA (Figure 3B). Cells were allowed to migrate toward 10% FBS as a chemoattractant similar to previous reports for Boyden chamber assays [36,37,38,39,40]. Representative images of Boyden chamber inserts show less cell migration (stained in purple) when ASAH1 was knocked down (Figure 3C). Quantification of migration, reflected by crystal violet-stained cells, demonstrated significantly decreased migration compared to the non-targeting control (Figure 3D). To differentiate between growth and migration, cells were plated and received the same treatment as cells added to Boyden chamber assays for growth assays. As quantified by Cell Titer Glo, we observed a decrease in growth with the ASAH1 knockdown (Figure 3E). However, when migration was normalized to growth, there was a significant effect on migration (Figure 3F). ASAH1 was knocked down using a second ASAH1-targeted shRNA, but cell viability was greatly reduced with this construct, so we did not continue with the migration analysis (Supplementary Figure S3). Together, these data indicate that the genetic inhibition of ASAH1 decreases migration independent of growth.

### 3.4. Carmofur Decreased Migration of GBM Cells

**Figure 4:**
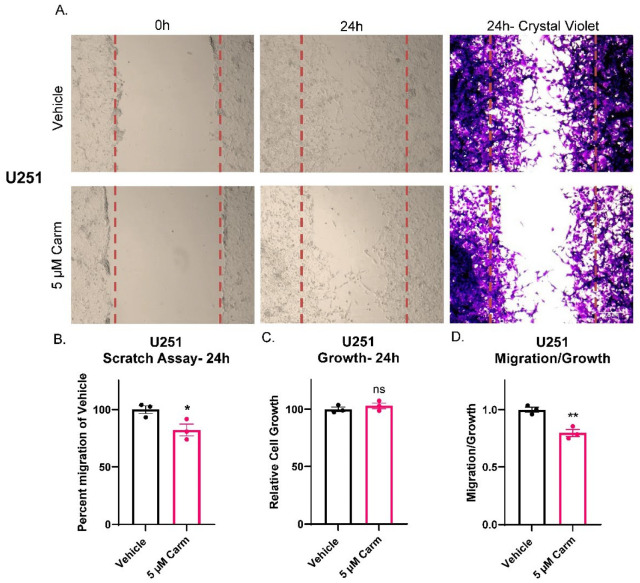
Carmofur decreased migration of U251 GBM cells. Cells were serum-starved in the presence of vehicle (DMSO) or 5 µM carmofur 16 h prior to introducing the scratch and re-treated at the time of the assay. After 24 h, cells were fixed in formalin and stained with crystal violet. (**A**) Representative images of U251 scratch assays at 0 h, 24 h, and 24 h with crystal violet staining taken at 10× magnification. (**B**) Image centers of 24 h 20× images were quantified using particle analysis in ImageJ and normalized to 0 h images before normalizing to vehicle for quantification. Comparison of groups was performed using independent *t*-test. (**C**) Matching growth was analyzed by Cell Titer Glo and normalized to vehicle control. (**D**) Percent migration was divided by the relative cell growth to determine the ratio of migration to growth. Data are displayed as means ± SEM. * *p* < 0.05, ** *p* < 0.01 with independent *t*-test (*n* = 3).

After confirming the link between ASAH1 expression and GBM migration, we identified commonly used inhibitors (carmofur, B13, and N-oleoylethanolamide) of ASAH1 and sought to determine the optimal concentration at which these inhibitors induce cell death in each cell line. We evaluated growth at seven days in the absence or presence of the three inhibitors in a standard cell line (U251) and/or cells derived from GBM PDXs (JX22 and D456) (Supplementary Figure S4). Of the three inhibitors, only carmofur and B13 significantly decreased growth at seven days. Guided by these findings, we next evaluated the effects of carmofur on migration. Carmofur was chosen as it is known to cross the blood–brain barrier and has been clinically used to treat other malignancies [11,41]. Using the IC_50_ values defined in the growth assays at 7 days, we assessed migration at earlier timepoints where growth was not affected. While carmofur is a derivative of 5-FU, a thymidylate synthase inhibitor, the concentration at which we treated the GBM cells is 100× less than the IC_50_ for thymidylate synthase inhibition [9]. Migration was assessed by scratch assays using the U251 GBM cells, as they grow in adherent monolayers in the presence of FBS even for short periods of time. Within 24 h, significant decreases in migration were evident with carmofur inhibition as shown in representative images (Figure 4A). Changes in migration were quantified by counting cells in the scratch after 24 h, with the scratch area being defined at time 0 (lines outlining area shown in red) (Figure 4B). Growth was unchanged in matching growth analysis performed with Cell Titer Glo assays (Figure 4C). As with the ASAH1 KD, the normalization of migration to growth showed migration effects of carmofur that did not depend on changes in growth (Figure 4D). We further confirmed this phenotype with a second ASAH1 inhibitor, B13 [42], which significantly decreased migration when normalized for growth (Supplementary Figure S5). While ASAH1 expression has been associated with the CD133^+^ stem-like population of GBM [8], we saw no changes in ASAH1 protein expression after 48 h of culture in FBS which is the maximum amount of time GBM cells were exposed to FBS in any experiment (Supplementary Figure S6).

### 3.5. Carmofur Decreased Migration of PDX-Derived GBM Cells

**Figure 5:**
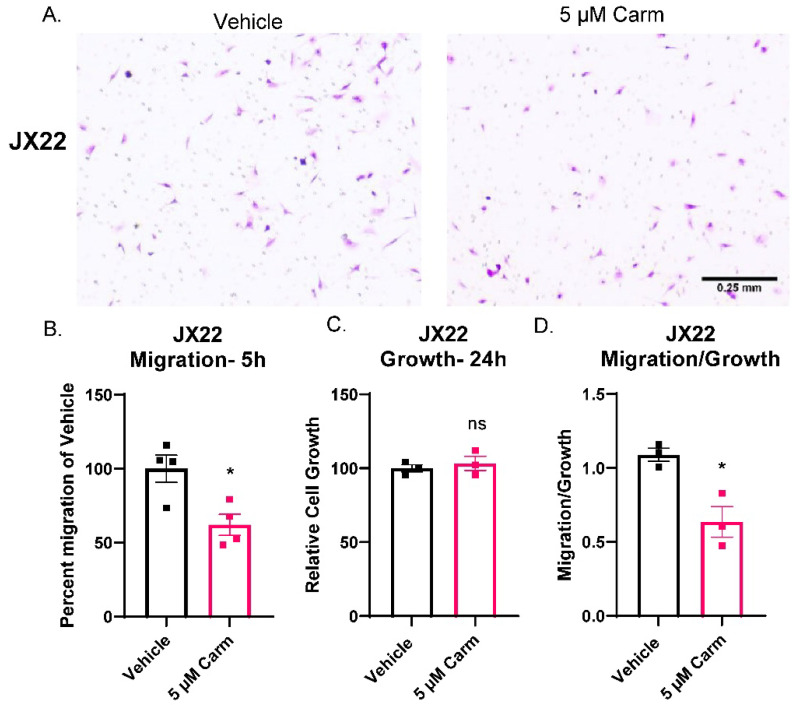
Carmofur decreased migration of PDX-derived GBM cells. Cells were serum-starved in the presence of vehicle (DMSO) or 5 µM carmofur 16 h prior to migration assay. At the time of assay, cells were plated at 50,000 cells/insert for the Boyden chamber assay on top of BTIC with 2× EGF/FGF and 2% FBS as the chemoattractant. At the same time, cells were plated at 5000 cells/well for matching growth assay. (**A**) Representative images of crystal violet-stained migration inserts from carmofur-treated JX22 GBM cell migration in Boyden chamber assays are shown. (**B**) Images were quantified using the cell count function on PICO Imagepress. (**C**) Matching growth was analyzed by Cell Titer Glo and normalized to vehicle control. (**D**) Percent migration was divided by the relative cell growth to determine the ratio of migration to growth. Data are displayed as means ± SEM. Not significant (ns), * *p* < 0.05 with independent *t*-test (*n* = 3–4).

After confirmation of migratory effects in a GBM cell line, we sought to confirm the effect of carmofur on cells isolated from the GBM PDX, JX22. Unlike the experiments shown in Figure 4, the JX22s were not cultured in FBS to maintain stemness during the time of the assay, and the chemoattractant was 2× EGF/FGF and 2% FBS media [17]. Additionally, the JX22 cells represent the mesenchymal GBM subtype which is known for its highly invasive and migratory phenotype [35,43]. As with shRNA knockdown of *ASAH1*, chemical inhibition of ASAH1 with carmofur blocked migration of GBM PDX-derived cells in Boyden chamber assays as shown by decreased purple staining in the carmofur-treated group (Figure 5A). Quantification of cells on Boyden chamber inserts revealed significantly decreased migration with the carmofur treatment (Figure 5B). We also analyzed invasion but did not see any significant decrease with the carmofur treatment (Supplementary Figure S7). Therefore, we concluded that ASAH1 inhibition decreased general cell motility not specific to invasion. Importantly, carmofur did not alter cell growth during the timeframe of the Boyden chamber assay (Figure 5C), and migration normalized to growth was significantly decreased (Figure 5D). Taken together, the observations presented in Figure 4 and Figure 5 suggest that carmofur decreases migration independent of growth effects in two separate GBM cell types and migration assays.

### 3.6. Pharmacologic Inhibition of ASAH1 Decreased Migration-Related Pathways

**Figure 6:**
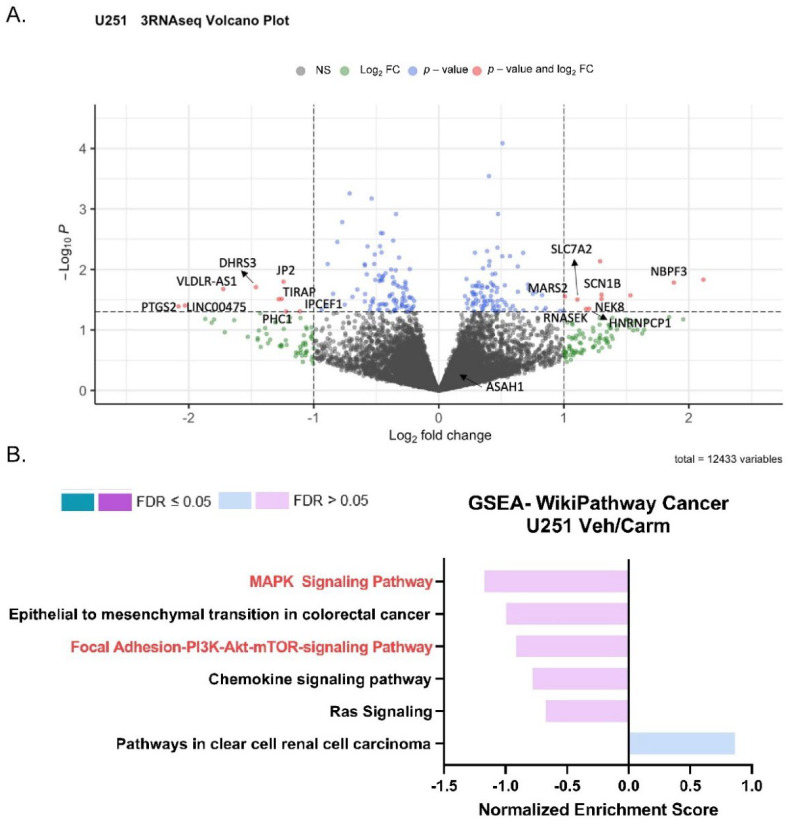
Pharmacologic inhibition of ASAH1 decreased migration-related pathways. To match conditions of the scratch assay, U251 cells were plated in 10% FBS, serum-starved in the presence of vehicle (DMSO) or 5 µM carmofur for 16 h, and changed to 10% FBS with new vehicle or carmofur for the final 24 h. Cells were collected for RNA-sequencing at the end of the 24 h treatment. (**A**) Visualization of RNA-sequencing by volcano plot of transcripts revealed that U251 cells treated with 5 µM carmofur displayed an altered pattern, relative to vehicle control (*n* = 3). (**B**) GSEA for genes with log_2_ FC > 0.5 or <−0.5 analyzed using WebGestalt (http://www.webgestalt.org) (accessed on 4 December 2021) and compared to Wikipathway cancer with a 3 gene minimum/pathway. Gene input included those with a base mean > 5 and a *p*-value < 0.2.

**Figure 7:**
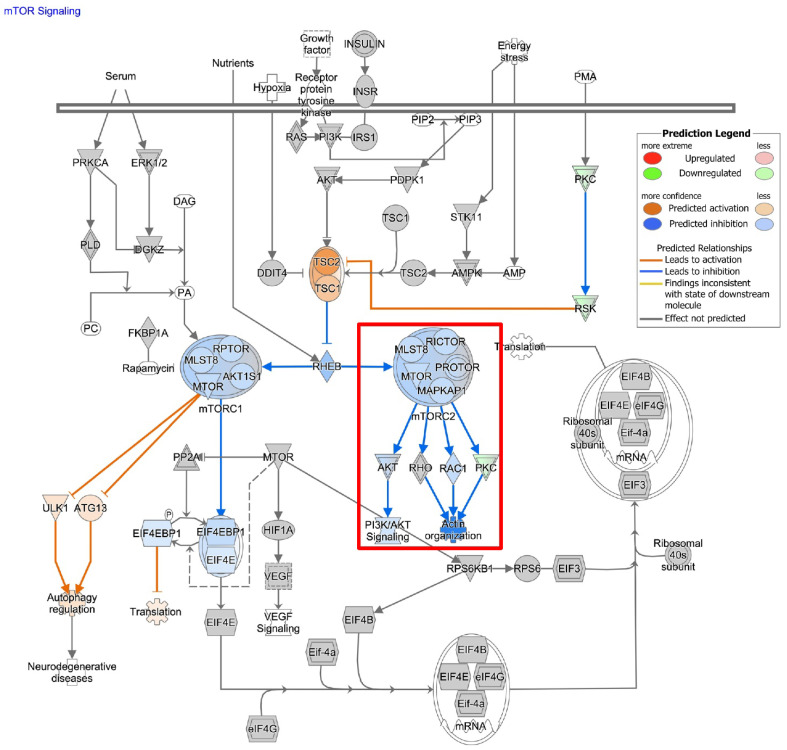
Carmofur treatment affects transcripts associated with mTOR signaling. Qiagen Ingenuity Pathway Analysis (https://digitalinsights.qiagen.com/IPA) (accessed on 18 February 2022) of RNA-sequencing data suggested decreases in mTOR signaling with potential downstream inhibition of PI3K/AKT signaling and Actin organization as highlighted in the red box.

To understand the impact of ASAH1 inhibition, we used RNA-sequencing and identified genes differentially expressed following the treatment with carmofur compared to vehicle control (Figure 6A). A total of 364 differentially expressed genes (*p* value < 0.2, base mean > 5) were identified. These genes were analyzed using WebGestalt [25] and WikiPathway Cancer functional databases for GSEA (Figure 6B) to identify pathways which are differentially expressed with the carmofur treatment. Based on normalized enrichment scores (NES), carmofur was found to decrease some of the same pathways identified in our correlation analyses of publicly available datasets (Figure 2). While not significantly different with carmofur treatment, pathways associated with migration, including MAPK signaling and focal adhesion-PI3K-Akt-mTOR signaling, may be altered (Table 2). Regulation of the assembly and disassembly of focal adhesions is required for migration of cancer cells [44]. Additionally, loss of focal adhesion kinase suppresses epidermal growth factor receptor signaling to decrease GBM cell migration [45]. To further identify relevant pathways altered by ASAH1 inhibition, we used Qiagen Ingenuity Pathway Analysis. Such analyses revealed “cancer” as the top disease or disorder (Supplementary Figure S8) and “cellular movement” as the top molecular and cellular function (Supplementary Figure S9) associated with the carmofur treatment. Of the network maps identified through this analysis, IPA predicted inhibition of mTOR signaling with downstream inhibition of PI3K/AKT signaling and actin organization Figure 7. Based on the current literature and our RNA-sequencing findings, we hypothesized that carmofur’s impact on GBM cell migration was mediated, at least in part, through either the MAPK or the PI3K-AKT-mTOR signaling pathway.

### 3.7. ASAH1 Inhibition Decreased pAKT, but Not pERK

**Figure 8:**
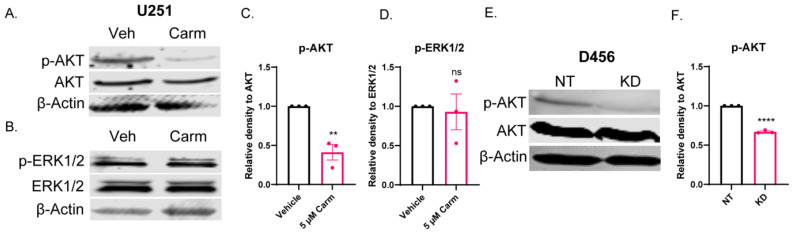
ASAH1 inhibition decreased pAKT, but not pERK. U251 cells were plated in 10% FBS followed by serum-starvation in the presence of vehicle (DMSO) or 5 µM carmofur for 16 h. Then, cells were stimulated with 10% FBS for 15 min followed by 45 min in the presence of fresh vehicle or carmofur. (**A**,**B**) Western blot expression of pAKT, AKT, pERK1/2, ERK1/2, and β-actin with carmofur treatment. (**C**,**D**) Densitometry values for each protein were determined using Image Studio Lite software. Quantification of densitometry values (*n* = 3) for expression of pAKT compared to total AKT and pERK1/2 compared to total ERK1/2. D456 cells were transduced with either non-targeting (NT) control or ASAH1 shRNA. After 48 h of transduction, cells were serum-starved for 16 h. Then, cells were stimulated with 10% FBS for 1 h. (**E**) Western blot expression of p-AKT, AKT, and β-actin for NT and ASAH1 KD cells. (**F**) Quantification of densitometry values (*n* = 3) for expression of p-AKT compared to total AKT. Data are displayed as means ± SEM. Not significant (ns), ** *p* < 0.01, **** *p* < 0.0001 with independent *t*-test.

Based on the observed migration phenotype and the pathways identified by the correlation analysis, we sought to determine whether AKT and/or ERK1/2 were involved in ASAH1-dependent migration. We assessed AKT and ERK1/2 phosphorylation and determined that pAKT was significantly decreased relative to total AKT (Figure 8A,C), but pERK1/2 was unchanged relative to total ERK1/2 (Figure 8B,D). Similarly, we assessed the ability of another ASAH1 inhibitor, B13, to inhibit AKT signaling and identified decreases in total AKT with the B13 treatment (Supplementary Figure S10). Finally, we confirmed that the shRNA knockdown of *ASAH1* decreased pAKT relative to total AKT (Figure 8E,F). We also explored the ability of carmofur to inhibit phosphorylation of focal adhesion kinase based on the pathway analysis, but we saw no changes at the protein level (Supplementary Figure S7). Our data suggest that ASAH1 modulates migration via changes in AKT signaling that does not depend on changes in ERK1/2 or focal adhesion kinase phosphorylation. Importantly, similar effects of ASAH1 expression on AKT signaling were observed using both pharmacologic and genetic approaches.

## 4. Discussion

Our study suggests that ASAH1 is overexpressed in GBM patients, and elevated expression of ASAH1 is associated with worse survival in IDH1-wildtype GBM patients. Targeting this group of glioma patients is key to extending survival as these tumors almost always recur despite aggressive therapies [3,46,47]. Further analysis of ASAH1 expression data suggested that ASAH1 regulates GBM cell migration through PI3K-AKT-mTOR signaling. We confirmed that ASAH1 inhibition decreased GBM cell migration and AKT signaling. We targeted ASAH1 genetically with shRNA and pharmacologically with carmofur, although there may be ASAH1-independent effects of carmofur that contribute to its effects. Inhibition of ASAH1 to decrease migration of GBM cells is a novel finding for both sphingolipid biology and neuro-oncology research.

Although our work has yet to identify the precise downstream mechanisms through which ASAH1-mediated decreases in AKT signaling affect migration, we hypothesize that AKT-mediated alterations of cytoskeleton-related proteins may be involved. For example, AKT signaling can induce expression or activation of GSK3β, F-actin, and Girdin [48,49,50,51]. Additionally, AKT1 has been shown to promote migration of fibrosarcoma cells through NF-κB [52]. This signaling mechanism as a means to decrease GBM migration was strengthened by the use of two different ASAH1 inhibitors as well as shRNA knockdown of ASAH1. Additional mechanistic approaches could be strengthened by using PDX-derived cells, samples from PDX treated with inhibitors in vivo, and/or increasing replicates to improve statistical power in the RNA-sequencing studies as well as implementing rescue experiments including the use of a constitutively active AKT construct [53] or overexpression of one of the potential downstream targets mentioned above. Moreover, assessment of migration ability could be conducted using spheroid migration assays to eliminate the use of FBS [54]. Overall, our work suggests that ASAH1 inhibition by shRNA or carmofur exerts its anti-migratory effects through decreased AKT signaling.

While direct inhibitors of AKT function have been tested in vitro, none have been efficacious in GBM patients [55]. One such inhibitor, perifosine, showed pre-clinical promise, but had limited blood–brain barrier penetrance, a significant challenge for any GBM therapeutic [56]. Ceranib-2, another acid ceramidase inhibitor, has been evaluated in breast cancer cell lines and decreased AKT signaling [57] as we have suggested in GBM cells with carmofur. However, carmofur has an advantage in that it is known to cross the blood–brain barrier [9]. Additionally, carmofur may decrease cell migration via both AKT and S1P signaling through inhibition of ASAH1. Since ceramides activate protein phosphatases, they could also decrease activity of JAK2, STAT5, and MYC [58]. In contrast to our findings, previous work in melanoma cells suggested that inhibiting ASAH1 increased pFAK. They further showed decreased invasiveness through activation of the integrin αVβ5-focal adhesion kinase signaling [59]. Therefore, the mechanism through which ASAH1 inhibition decreases migration may be specific to certain cancer types. While not explored in this study, the ability of carmofur to decrease chemokine signaling may serve as an added benefit for therapeutic combinations. However, a potential concern of targeting the sphingolipid balance is that the modulation of S1P receptor 1 could worsen lymphopenia in brain tumor patients [60]. Targeting ASAH1 should increase ceramides and decrease S1P without affecting the S1P receptors, but the effect on immune suppression in these patients would need to be considered for future clinical trials.

Restoration of the sphingolipid balance has long been proposed as a mechanism to attenuate multiple hallmarks of cancer including proliferation, migration, and angiogenesis [61]. As highlighted in our previous review, attempts at modulating the sphingolipid balance in brain tumor patients have not yet proven to be successful [5]. Recent work in brain tumors has focused on inhibiting sphingosine kinase 1 to decrease the production of S1P, thus preserving the pro-apoptotic capability of ceramides. Targeting sphingosine kinase 1 in GBM cell lines decreased angiogenesis along with decrease in growth of temozolomide-resistant GBM cells [1,62]. Unfortunately, these inhibitors have not yet been tested in GBM patients. Based on the lack of FDA-approved sphingosine kinase inhibitors for GBM and the finding that ASAH1 is increased following radiotherapy, we chose to focus on ASAH1, an enzyme responsible for producing sphingosine and regulating ceramide catabolism [4,63]. This approach may preferentially increase ceramides compared to inhibiting sphingosine kinases as these inhibitors have been suggested as combination or maintenance therapy following another ceramide-inducing agent [64].

While this study did not directly compare the effects of 5-FU to carmofur for assessment of migration, 5-FU has a low therapeutic index when delivered systemically and has not been studied for its anti-migratory potential in GBM [65]. In contrast to 5-FU, carmofur is more orally bioavailable and generally well-tolerated by patients even when given multiple times per day to overcome the short half-life [41]. Therefore, carmofur may have dual roles as a 5-FU pro-drug and a modulator of the sphingolipid balance in GBM. As modulation of the sphingolipid balance has emerged as an important target for GBM therapeutics, more inhibitors are being developed in addition to the identification of previously unknown mechanisms for current drugs. For example, tamoxifen, a well-established treatment of ER-positive breast cancer, was recently shown to inhibit ASAH1 re-enforcing the utility of repurposed drugs for GBM patients [66]. Taken together, we propose the use of ASAH1 inhibition, specifically by carmofur, to decrease the migration of GBM cells. Finally, inhibiting the migration of cancer cells by blocking ASAH1 may be a promising avenue for metastatic brain tumors [18,67,68,69,70].

## Figures and Tables

**Table 1:** The Focal Adhesion-PI3K-AKT-mTOR pathway appeared in all three *ASAH1* correlation analyses. Data represent enrichment scores (ES), normalized enrichment scores (NES), *p* values, and false discovery rate (FDR) for the Focal Adhesion-PI3K-AKT-mTOR pathway in each dataset.

Data Set	Gene Set	ES	NES	*p* Value	FDR
Gravandeel	Focal Adhesion-PI3K-Akt-mTOR-signaling pathway	0.2472	2.0613	0.00351	0.03479
TCGA GBM HG-U133A		0.1867	0.9670	0.49793	0.48293
TCGA GBM Agilent-4502A		0.2265	1.3467	0.14067	0.32006

**Table 2:** Pharmacologic inhibition of ASAH1 suggested decreases in migration-related pathways. Table for GSEA analysis including ES, NES, *p* values, and FDRs for each pathway as well as differentially expressed genes (DEGs) for that pathway.

Gene Set	Description	Size	Leading Edge Number	ES	NES	*p* Value	FDR	DEGs
WP382	MAPK Signaling Pathway	4	4	−0.5072	−1.1697	0.2591	1	*MAPK12, PLA2G4A, PDGFB, DUSP7*
WP4239	Epithelial to mesenchymal transition in colorectal cancer	3	2	−0.4992	−0.9973	0.4734	1	*COL4A3, MAPK12*
WP3932	Focal Adhesion-PI3K-Akt-mTOR-signaling pathway	5	5	−0.3613	−0.9129	0.5568	0.9598	*ITGA7, ANGPT1, SREBF1, GNG10, PDGFB*
WP3929	Chemokine signaling pathway	3	3	−0.3822	−0.7803	0.7407	0.9453	*CCL26, GRK5, GNG10*
WP4223	Ras Signaling	3	3	−0.3362	−0.6739	0.9022	0.876	*KSR1, PLA2G4A, GNG10*
WP4018	Pathways in clear cell renal cell carcinoma	4	4	0.3862	0.8617	0.6248	0.6455	*KSR1, PDGFB, PSAT1, PHGDH*

## Data Availability

Data for RNA-sequencing analysis can be found on the Gene Expression Omnibus with the accession number GSE179087.

## Supplementary Materials

The following are available online at https://www.mdpi.com/article/10.3390/cells11121873/s1, Figure S1: Survival Curves for ceramidase expression in Low- and High-grade gliomas, Figure S2: GSEA tables for *ASAH1* correlations, Figure S3: Growth analysis with ASAH1 shRNA #2, Figure S4: Growth analysis with ASAH1 inhibitors, Figure S5: Migration analysis with B13, Figure S6: ASAH1 expression with FBS media, Figure S7: Invasion and phospho-FAK analysis, Figure S8: Diseases and disorders from IPA, Figure S9: Molecular and cellular functions from IPA, Figure S10: AKT expression with B13, Figure S11: Full-sized blots for Figure 8, Figure S12: Full-sized blots for supplemental figures. Replicates for each experiment are available upon request.
